# Characterization, Spatial Variation and Management Strategy of Sewer Sediments Collected from Combined Sewer System: A Case Study in Longgang District, Shenzhen

**DOI:** 10.3390/ijerph18147687

**Published:** 2021-07-20

**Authors:** Yongpeng Luo, Shenxu Bao, Siyuan Yang, Yimin Zhang, Yang Ping, Chao Lin, Pan Yang

**Affiliations:** 1 School of Resources and Environmental Engineering, Wuhan University of Technology, Wuhan 430070, China; luoyp@whut.edu.cn (Y.L.); siyuan.yang@whut.edu.cn (S.Y.); zym126135@126.com (Y.Z.); 2 State Environmental Protection Key Laboratory of Mineral Metallurgical Resources Utilization and Pollution Control, Wuhan University of Science and Technology, Wuhan 430081, China; 3 Shenzhen Water Planning and Design Institute Co., Ltd., Shenzhen 518116, China; pingy@swpdi.com (Y.P.); linc@swpdi.com (C.L.); 4 Department of Civil and Environmental Engineering, University of Illinois at Urbana Champaign, 205 N Mathews Ave, Urbana, IL 61820, USA; pyangac@illinois.edu

**Keywords:** sewer sediment, combined sewer system, hazardous contaminants, spatial distribution, municipal sludge management

## Abstract

In the urban drainage system, the formation of sewer sediments is inevitable, and the removal of sewer sediments is necessary for system maintenance. Disposal of arisings from sewer sediment removal is becoming a serious environmental issue. The current knowledge of sewer sediments is limited, which is restrained to sewer sediments management. To better understand this municipal waste, the sewer sediments of a combined sewer system in Longgang District, Shenzhen were collected and characterized, and the spatial distribution characteristics of contaminants were analyzed. Based on the bivariate correlation analysis, it is found that many contaminants in sewer sediments have a strong relationship with spatial variables. Compared to the sewer sediments in industrial areas, those in residential areas contain higher concentrations of Hg and phosphorus. The sediments in the sewage conduit also contain more organic matter (OM), phosphorus, Cu, and Ni, and the sediments in the rainwater conduit contain a higher concentration of Cd. Moreover, the sediments produced in different catchments also show huge differences in the content of contaminants. These spatial distribution characteristics may provide help for the further classification of sewer sediments, thereby making the disposal of sediments more targeted. According to the local standards of sludge disposal, land application and incineration are not suitable for managing sewer sediments due to the low OM content and poor lower heating value (LHV). Although sanitary landfill is feasible for sewer sediments disposal, the complicated composition of sewer sediments still poses the risk of polluting the surrounding environment. The management of sewer sediments via the production of building materials is a promising technical route that can avoid the migration of hazardous contaminants and produce valuable products. This study may improve our understanding of sewer sediments and provide a reliable recommendation for sewer sediment management.

## 1. Introduction

Combined sewer systems (CSS) are widely used worldwide to collect and transfer rainwater runoff, domestic sewage, and industrial effluents generated in cities [1,2]. With the rainwater runoff washing and daily municipal sweeping, the dust-and-dirt of the land surface is drained via street inlets into CSS. In addition, the domestic sewage and industrial effluents discharged into the CSS also carried many suspended solids. During the long-term service of CSS, these in-sewer solids are gradually deposited on the drainage channel bottom and form the sewer sediments. In the United States, it is estimated that 5% to 30% of suspended solids and pollution loads in daily sewage are deposited [3]. In Europe, the deposition rates of CSS are measured from 30 to 500 g/(m∙d) [4].

The accumulation of sewer sediments leads to a decrease in flow-carrying capacity of the waterway and even complete blockage, which may cause an upstream surcharge [5], local flooding [6]. Under the microbial action, the sewer sediments will release a lot of odors and gas (e.g., hydrogen sulfide and methane) [7,8,9], which threatens the health of city dwellers. The corrosive substances also related to microbial metabolism will corrode the inner wall of the pipeline and shorten the pipeline service life [10]. In addition, the presence of sewer sediments also causes a series of problems in the combined sewer overflows (CSO) control. During the storm events, the resuspension of sewer sediments would dramatically aggravate flow pollution and causes failure to meet discharge standards. It is reported that 80% of pollution loads of CSO are originated from sewer sediments [11]. Especially in the wet-weather season, once the wastewater treatment plant is overloaded, the untreated CSO will be run into the surrounding water body and causes serious environmental issues.

Therefore, it is necessary to clear sewer sediments regularly for the maintenance of CSS. Flushing is a common method for sewer cleaning, which is to create an artificial heavy flow by either quickly pouring external water or rapidly opening a water restraining gate. However, the cleaning efficiency of traditional manual flushing is limited by the conduit characteristics, such as shape, diameter, length, slope, etc. [12]. The flushing rate should be controlled so that the CSO not be produced at downstream nodes. Moreover, the migration of sewer sediments caused by flushing will affect the operation of downstream facilities such as water storage tanks and pumping stations. In recent years, sewage suction trucks with vacuum flushing systems have gradually been applied in many cities, and it could generate a cleaning wave by equipping vacuum pumps and suck sewer sediments out of the sewer. The sewage suction truck can directly remove the sediments at the deposition sites instead of flushing the sewer sediments downstream, which avoids adverse effects on downstream facilities.

With the popularity of sewage suction trucks, the amount of sewer sediment that needs to be disposed of has gradually increased, and the management of sewer sediments has become a severe challenge for urban development. Li et al. [13] investigated illicit drugs and pharmaceuticals in sewer sediments and found that most drug compounds are difficult to degrade under natural pH conditions. In some Eastern European countries, researchers have found that the sewer sediments contain relatively high pollution levels of phthalate acid esters (PAEs), which pose potential risks to hydrobiots and human health [14]. According to Li et al.’s [15] assessment of heavy metal pollution in sewer sediments in central Beijing, China, most of the heavy metals in the sediments are ranked at “low risk” levels, while cadmium fell into the “high to very high risk” category. Although the environmental risks of many pollutants presented in sewer sediments are very clear, the sewer sediments are still not properly disposed of. Due to the lack of systematic and comprehensive investigations on the characteristics of sediments, there are still many difficulties in the collection and classification of sediments, which limits the disposal and utilization of sewer sediments. It is urgent to improve our basic understanding of sewer sediment and find a proper technical route for sewer sediment management.

In this study, a large number of sewer sediments samples were collected and characterized as comprehensively as possible. The characteristics of sewer sediments under different spatial distributions were analyzed, which may provide a reasonable basis for the collection and classification of sewer sediments. The difference between sewer sediment and common municipal sludge was discussed, and the feasibility of technical routes for sewer sediment management was evaluated. It may improve our understanding of sewer sediments and provide a reliable recommendation for sewer sediment disposal.

## 2. Materials and Methods

### 2.1. Study Site

**Figure 1:**
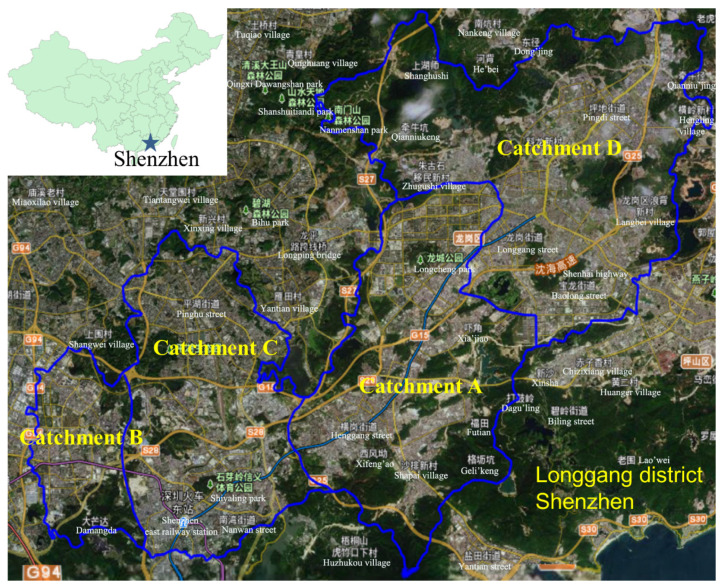
Study site.

This study was conducted in Longgang district, Shenzhen City, Guangdong province, China. As shown in Figure 1, the Longgang district can be delineated into four catchments based on elevation. The land-use types of deposition sites include industrial areas (IA) and residential areas (RA). The CSS of the Longgang district contains four types of drainage channels, including box culverts (BC), storm sewer conduits (SSC), sewage conduits (SC), and combined conduits (CC). A total of 73 samples of sewer sediments are collected from the CSS. The sample numbers and corresponding spatial characteristics are given in Table S1 in the Supplementary Information.

### 2.2. Sample Collection and Characterization

The collection method, parameters measurement, and chemical pollutants detection of sewer sediments are according to the National Standards of the People’s Republic of China (GB 24188-2009) [16]. All the samples are taken out from the deposition site in the pipeline by shovel or sewage suction truck. Gravel, plastic, shells, and animal and plant debris in the sample were manually removed. After the samples were drained as much water as possible, they were packed in plastic bags or glass bottles, and the mass of each sample is greater than 2 kg.

According to the determination method of CJ/T 221-2005 [17], sewer sediment parameters were measured, including pH value, bulk density, moisture content, lower heating value (LHV), and ash content. Also, sewer sediments were analyzed for various chemical pollutants, including Zn, Cu, Ni, As, Cd, Cr, Hg, Pb, total nitrogen (TN), total phosphorous (TP), total potassium (TK), organic matter (OM), sulfide, petroleum oil (PO), adsorbable organic halides (AOX), cyanides, and volatile phenols (VP).

The pH was measured using a pH electrode (PHS-3C, Leiz, Nanjing, China). The LHV of sewer sediment was determined by using a differential scanning calorimeter (TGA/DSC3+, Mettler Toledo, Greifensee, Switzerland). Bulk density was measured by weighing the mass of sewer sediment with a known volume. The sewer sediment is dried to constant weight at 105 °C, and then ignition at 550 °C. The moisture content, OM content, and ash content of the sewer sediment were calculated by Equations (1)–(3):

Moisture content = (*m* − *m*_105 °C_)/*m* (1)

OM = (*m*_105 °C_ − *m*_550 °C_)/*m*_105 °C_ (2)

Ash content = *m*_550 °C_/*m*_105 °C_ (3)

where *m*, *m*_105 °C_ and *m*_550 °C_ are the initial mass of samples, the mass after drying at 105 °C and the mass after ignition at 550 °C, respectively.

TN was analyzed using a Kjeldahl analyzer (Kjeltec 8100, FOSS, Hilleroed, KH, Denmark). TP and TK were measured using the spectrophotometric method and the flame photometry method, respectively. Sulfide, PO, AOX, VP, and cyanides were determined by using the spectrophotometric method.

For heavy metals analysis, 0.1 g of dried sewer sediment was digested in a Teflon digestion vessel with a 10 mL mixture of HNO_3_, HF, H_2_O_2_, and HCl with a ratio of 2:2:2:6. Then, they were treated in a microwave digestion system (Anton Paar, Graz, Austria) for 10 min until reaching 165 °C and were kept at this temperature for 20 min. After cooling, the extractions were filtered and made up to 25 mL with ultrapure water. The concentrations of Zn, Cu, Ni, As, Cd, Cr, Hg, and Pb were determined by inductively coupled plasma spectrometry (820-MS, Varian, Lake County, IL, USA).

### 2.3. Correlation Analysis

To explore the relationship among the different variables related to sewer sediments, a bivariate correlation analysis was carried out. The significance levels were evaluated by Spearman’s rank correlation coefficient (*ρ*), and the formula is as follow:

(4) $$\rho =1-\frac{6\sum_{i}{\left(R_{1i}-R_{2i}\right)}^{2}}{n\left(n^{2}-1\right)}$$

where *n* is the number of pairs of observations, and *R*_1*i*_ is the first set of data, and *R*_2*i*_ is the second set of data. A *ρ* value of +1 means a perfect positive association of those two variables, and a *ρ* value of 0 means no association of those two variables, and a *ρ* value of −1 means a perfect negative association of those two variables. The closer the *ρ* value to 0, the weaker the association between those two variables.

## 3. Results

### 3.1. Bulk Density, Moisture Content, and pH Value

**Figure 2:**
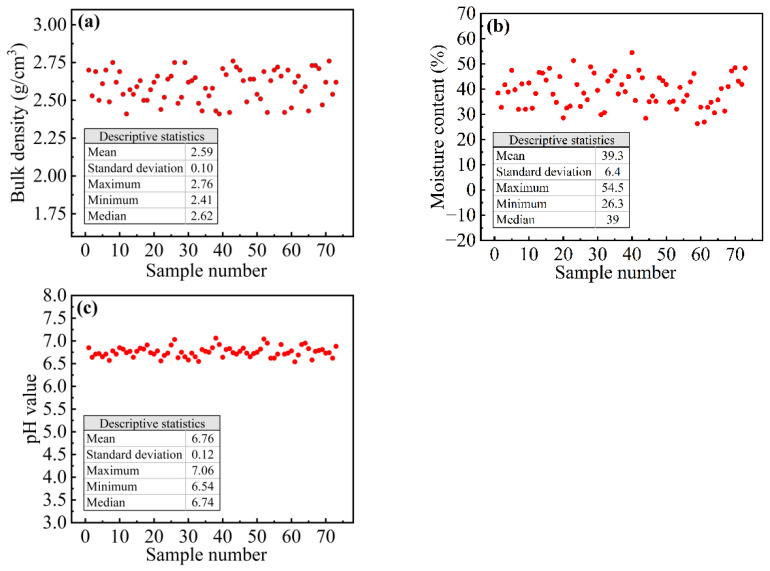
(**a**) Bulk density, (**b**) moisture content, and (**c**) pH value of sewer sediment samples.

The bulk density, moisture content, and pH value of the sewer sediment samples are presented in Figure 2. As shown in Figure 2a, the density of sewer sediments is between 2.41 and 2.76 g∙cm^−3^, the median density is 2.62 g∙cm^−3^, and the average density is 2.58 g∙cm^−3^ with a standard deviation of 0.10 g∙cm^−3^. Among these sediment samples, the highest moisture content is 54.5%, the lowest moisture content is 26.3%, the median moisture content is 39.0%, and the average moisture content is 39.3% (Figure 2b). The pH value of sewer sediments is distributed in a narrow range of 6.54 to 7.06. The median pH value of the sample is 6.74, the mean is 6.76, and the standard deviation is only 0.12 (Figure 2c).

### 3.2. Particle Size Distribution, LHV, and Ash Content

**Figure 3:**
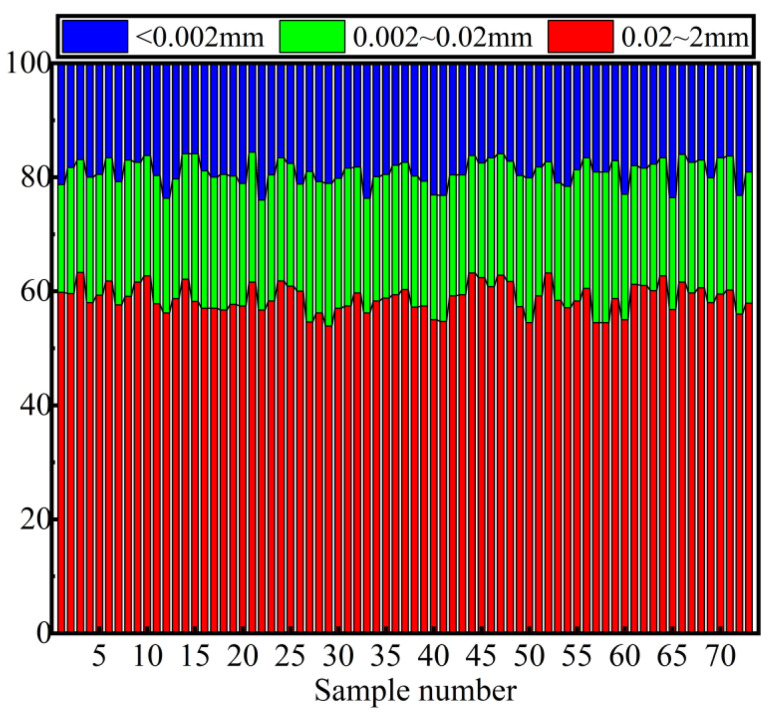
Particle size distribution of sewer sediment samples.

According to the size, the particles of sewer sediments can be classified into the sand, silt, and clay, and the corresponding particle size ranges are 0.2 to 0.02 mm, 0.002 to 0.02 mm, and less than 0.002 mm, respectively. From Figure 3, it is clear that all samples present a similar particle size distribution. Sand is the most abundant particle in sediments, followed by silt and clay, and the average content is 58.81%, 22.14%, and 19.05%, respectively. In terms of sand content in sewer sediments, the highest content is 63.3%, and the lowest content is 53.9%. The contents of silt and clay are range from 18.8% to 26.4%, and 15.6% to 24.0%, respectively.

**Figure 4:**
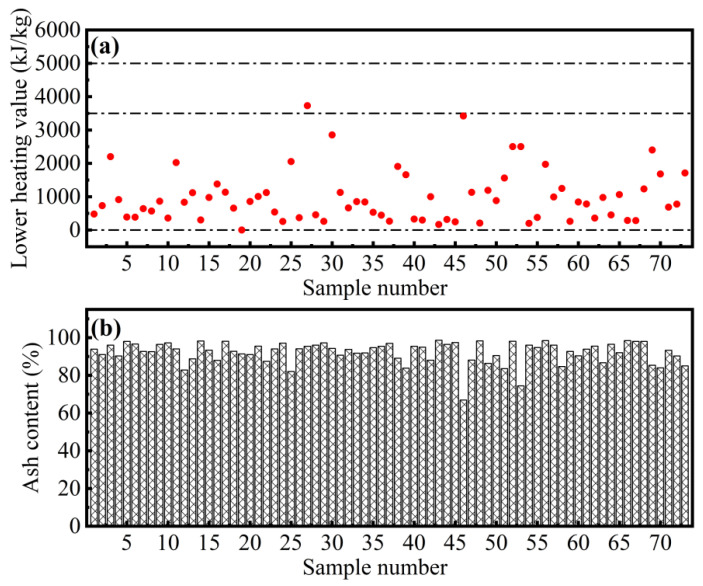
(**a**) LHV and (**b**) ash content of sewer sediment samples.

As shown in Figure 4, the highest LHV of 3731 kJ∙kg^−1^ is measured, the lowest LHV is 0 kJ∙kg^−1^, the median LHV is 840 kJ∙kg^−1^, and the mean is 987 kJ∙kg^−1^. The LHV of all samples is less than kJ∙kg^−1^, only about 3% of the samples have LHV close to 3500 kJ∙kg^−1^, and the rest of the samples have LHV less than 3000 kJ∙kg^−1^ (Figure 4a). The sewer sediments have high ash content, where the mean is as high as 92.20%, the median reaches 93.86%, the maximum of 98.59% is reached, and the minimum is 66.87%. (Figure 4b).

### 3.3. OM, TN, TP, and TK Content

**Figure 5:**
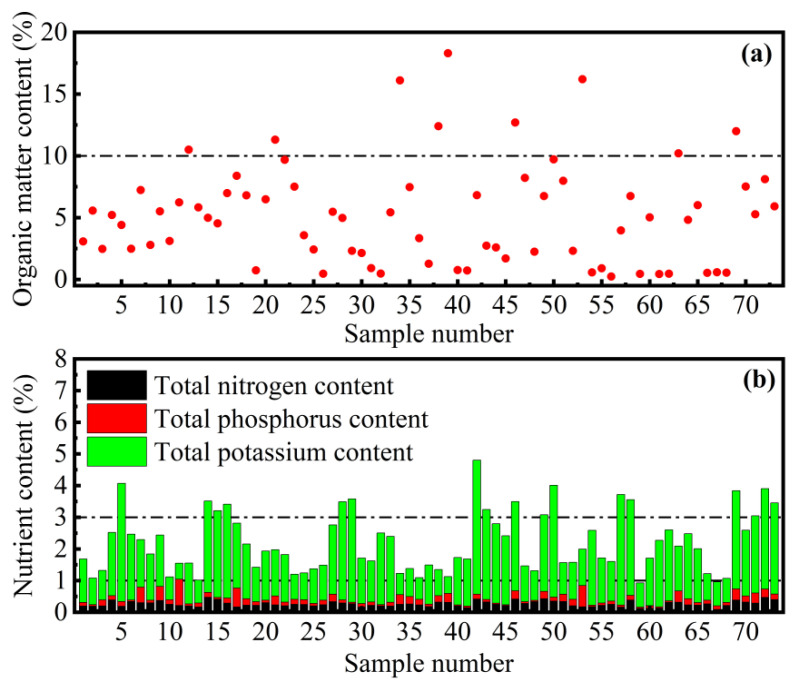
(**a**) OM content and (**b**) nutrient content of sewer sediment samples.

As shown in Figure 5, the OM content of sewer sediments ranges from 0.24% to 18.30%, and the median is 4.99%, and the mean is 5.21% with a standard deviation of 4.10%. Among these samples, sewer sludge with OM content greater than 10% accounted for 12.3% (Figure 5a). Meanwhile, there are some nutrients in sewer sediments, including potassium, nitrogen, and phosphorus. The contents of TK, TN, and TP in the sediments are in the range of 5040 mg∙kg^−1^ to 42,300 mg∙kg^−1^, 877 mg∙kg^−1^ to 4710 mg∙kg^−1^, and 279 mg∙kg^−1^ to 8300 mg∙kg^−1^, respectively. The median and mean of total potassium content are 15,000 mg∙kg^−1^ and 17,711 mg∙kg^−1^, the median and mean of total nitrogen content are 2480 mg∙kg^−1^ and 2618 mg∙kg^−1^, and the median and mean of total phosphorus content are 1230 mg∙kg^−1^ and 1690 mg∙kg^−1^ (Figure 5a). Moreover, the total nutrient content of all samples is greater than 1%, and 23.3% of the samples have total nutrient content greater than 3%.

### 3.4. Hazardous Pollutants

The contents of various contaminants in the sediments are measured, and the results are given in Table S2 in Supplementary Information. There are many heavy metals present in sewer sediments, including Zn, Cu, Ni, As, Cd, Cr, Hg, and Pb. Zn is the most abundant heavy metal in the sewer sediment, with an average concentration of 649.79 mg∙kg^−1^. The average concentrations of other heavy metals are as follow Cu = 198.42 mg∙kg^−1^, Cr = 135.45 mg∙kg^−1^, Pb = 91.22 mg∙kg^−1^, Ni = 61.14 mg∙kg^−1^, As = 16.09 mg∙kg^−1^, Cd = 1.18 mg∙kg^−1^, Hg = 1.10 mg∙kg^−1^. Also, other hazardous substances contained in sewer sediments, sulfide, PO, AOX, cyanides, and VP are detected. The sulfide content range from 23.40 mg∙kg^−1^ to 54.70 mg∙kg^−1^, and the mean is 32.68 mg∙kg^−1^. The content of PO is between 188.42 mg∙kg^−1^ and 1101.34 mg∙kg^−1^, and the mean is 557.38 mg∙kg^−1^. The AOX content is in a wide range of 42.52 mg∙kg^−1^ to 1946.00 mg∙kg^−1^, with a mean of 511.22 mg∙kg^−1^. The average content of cyanides and VP are 0.07 mg∙kg^−1^ and 0.38 mg∙kg^−1^, respectively.

## 4. Discussion

### 4.1. Bivariate Correlation Analysis

**Figure 6:**
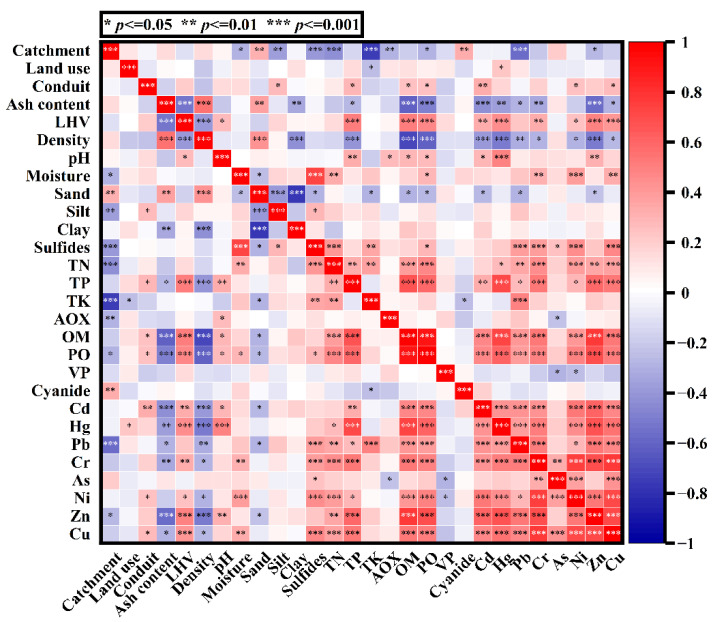
Correlation coefficient matrix of variables related to sewer sediment.

To understand the characteristics of the sediment as much as possible, the bivariate correlation analysis between the sediments-related variables was performed. According to Figure 6, it can be found that many components in the sediment show a close relationship with the sediment parameters. There is a positive correlation between the density and the sand content, and a negative correlation with the content of clay, OM, PO, TP. This indicates that sewer sediments with higher density always contain more sand and less clay, OM, and TP. It is consistent with the basic understanding that the more heavy substances (sand) and less light substances (clay, OM, TP, etc.) contained in the sediments, the greater the density of the sediments. In addition, the pH value is positively correlated with the concentration of some heavy metals (Zn, Hg, and Cd). This shows that the higher the pH value, the higher the concentration of Zn, Hg, and Cd in the sediments. It may be that the higher pH value causes more heavy metals to be transferred from the wastewater to the sediment by precipitation [18]. There is also a strong positive correlation between moisture content and sulfide content, which indicates that the higher the moisture content, the more sulfides contained in the sediments. It may be due to the dissolution and absorption of sulfide by water [19,20]. Moreover, the LHV is positively correlated with the content of PO, OM, and TP, while the ash content is negatively correlated with these variables. This indicates that the more PO, OM, and TP contained in the sediments, the more thermal energy released and the less fly ash produced after incineration. The OM, PO, TP content, and the concentrations of most heavy metals have a strong positive correlation with each other, which is probably due to the tendency of these heavy metals to be adsorbed on OM, PO, and TP [21]. Besides, many components in the sediments show a correlation with spatial variables (such as land uses, conduits, and catchments), indicating significant differences in the characteristics of sewer sediments at spatial distribution.

### 4.2. Spatial Distribution Characteristics

#### 4.2.1. Land Uses

**Figure 7:**
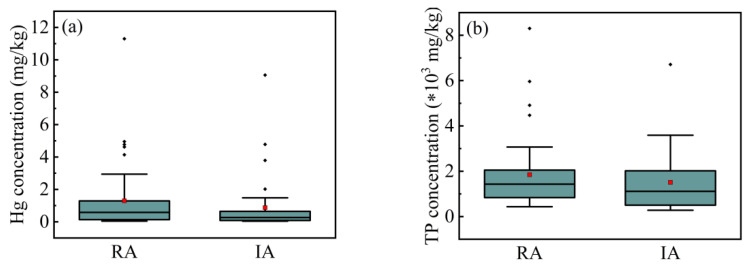
Box-plot of (**a**) Hg and (**b**) TP levels in different land uses. *: multiplication sign.

Land use is a variable that describes the main human activities in a specific area. According to the land use of the deposition site, the sediment samples collected in this study can be classified into RA samples and IA samples. In the RA, the mean and median of Hg contents are 1.30 mg∙kg^−1^ and 0.58 mg∙kg^−1^, respectively, while in the IA, the mean and median of Hg concentrations are 0.87 mg∙kg^−1^ and 0.26 mg∙kg^−1^, respectively (Figure 7a). Also, the sediments from RA have an average TP content of 1848 mg∙kg^−1^ and a median of 1430 mg∙kg^−1^, and the sediments from IA have an average TP content of 1509 mg∙kg^−1^ and a median of 1115 mg∙kg^−1^ (Figure 7b). The sewer sediments in RA contain more Hg and phosphorus than that in IA, this may show that human activities in Longgang District produce more phosphorus and Hg pollution than industrial production activities. Except for phosphorus and Hg, other components in sewer sediment do not show a correlation with land use types, which indicates that the distribution of these components in industrial and residential areas are similar.

#### 4.2.2. Conduits

**Figure 8:**
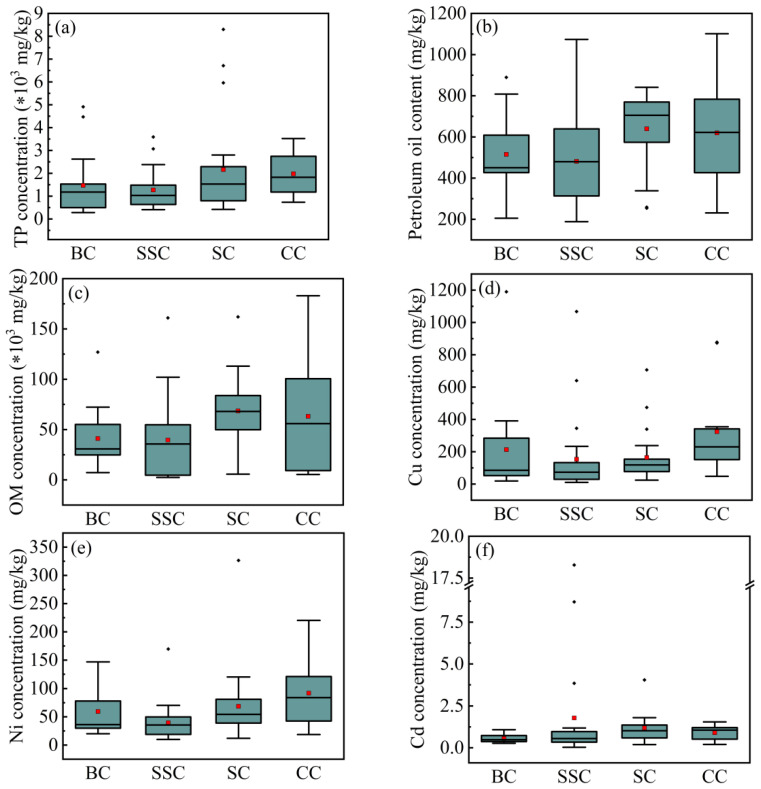
Box-plot of (**a**) TP, (**b**) PO, (**c**) OM, (**d**) Cu, (**e**) Ni, and (**f**) Cd level in different conduits. *: multiplication sign.

Sewer sediment originates from wastewaters, and these various wastewaters are transported by different types of conduit. Generally speaking, the rainwater is main transferred by BC and SSC, and the domestic sewage and industrial effluents are transferred by SC. In addition, some rainwater and sewage in the CSS will share the CC for transportation. Hence, the composition characteristics of sewer sediments usually vary with the conduits type. The contents of TP, OM, PO, and the concentrations of Cu, Ni, and Cd exhibit a correlation with the conduit variables, respectively (Figure 6). It means that these substances have a different distribution in the sewer sediments collected from different conduits. As shown in Figure 8a, the average TP content in the sediment of SC is 2163 mg∙kg^−1^, and the highest TP content among the samples of SC reaches 8300 mg∙kg^−1^, which is remarkably higher than the sediment in other conduits. The average TP content in the sediments of CC is also higher than that of BC and SSC sediments (CC = 1976 mg∙kg^−1^, BC = 1469 mg∙kg^−1^, SSC = 1272 mg∙kg^−1^). Also, the PO and OM have a similar distribution pattern to TP in various sediments, and both show higher contents in sewer sediments of SC and CC (Figure 8b,c). In addition, the sewer sediments with the highest average concentrations of Cu and Ni came from CC (Cu = 323.00 mg∙kg^−1^, Ni = 91.90 mg∙kg^−1^), followed by BC (Cu = 212.87 mg∙kg^−1^, Ni = 59.44 mg∙kg^−1^), SC (Cu = 164.27 mg∙kg^−1^, Ni = 68.63 mg∙kg^−1^) and SSC (Cu = 153.45 mg∙kg^−1^, Ni = 39.47 mg∙kg^−1^) (Figure 8d,e). The distribution of Cd is opposite to that of Cu and Ni. The average concentration of Cd in sediments of SSC is higher than that of other conduit sediments (SSC = 1.78 mg∙kg^−1^, SC = 1.19 mg∙kg^−1^, CC = 0.89 mg∙kg^−1^, BC = 0.55 mg∙kg^−1^), and the highest Cd concentration in samples collected in SSC even reached 18.28 mg∙kg^−1^ (Figure 8f).

The sediments of SC and CC contain more phosphorus, PO and OM, and the concentration of Cu and Ni are also higher. This is because the concentrations of phosphorus, PO and OM in domestic sewage and industrial effluents are remarkably higher than those in rainwater. In addition, due to the corrosion of pipelines, the concentration of Cu and Ni in sewage also is higher than that of rainwater. However, there is a higher concentration of Cd in sewer sediments of SSC. This may be due to rainwater converges on the ground to form surface runoff before entering the drainage system, and the surface runoff washes the ground and carries a large amount of Cd metal which is closely related to traffic activities [22,23].

#### 4.2.3. Catchments

**Figure 9:**
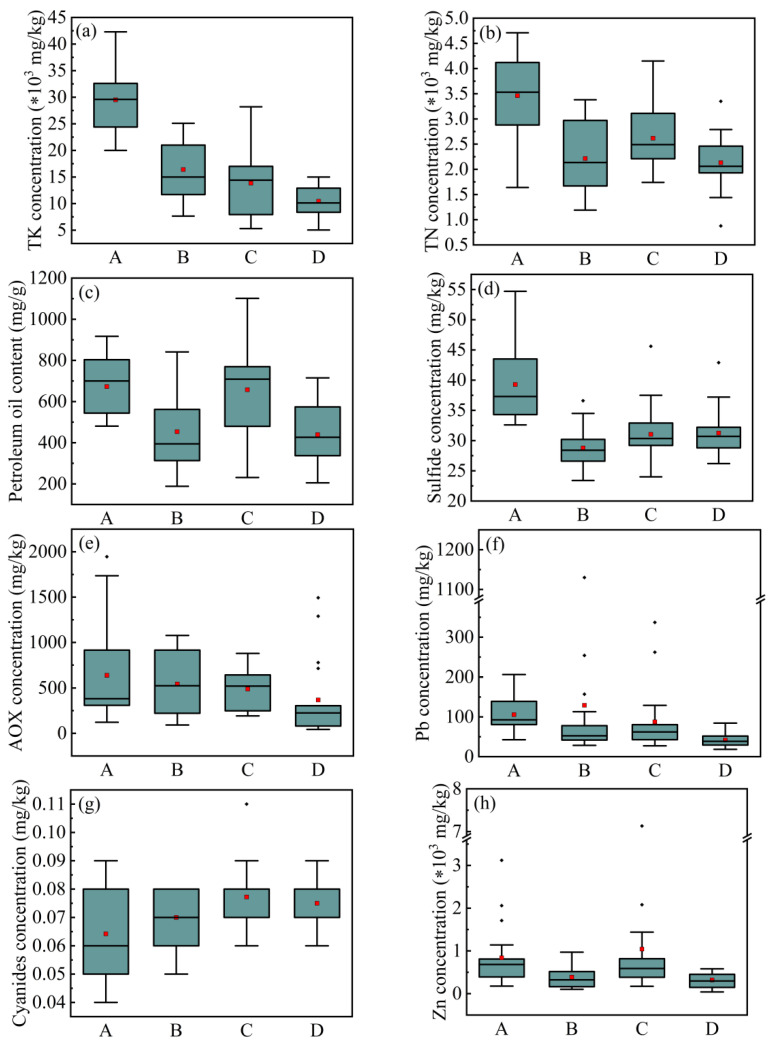
Box-plot of (**a**) TK, (**b**) TN, (**c**) PO, (**d**) sulfides, (**e**) AOX, (**f**) Pb, (**g**) cyanides, and (**h**) Zn level in different catchments. *: multiplication sign.

Catchments refer to the surface area through which surface runoff or other substances converge to a common outlet. There is a strong relationship between the catchment and the content of TK, and Pb, and a moderate correlation between the catchment with the content of TN, sulfides, AOX, PO, cyanides, and Zn (Figure 6). By comparing the TK content of sediments in different catchmentscatchments, it can be found that the TK content in the sediments of catchment A is much higher than that in the other catchments. The average TK content of sewer sediments in catchment A is 29,511 mg∙kg^−1^, while 16,402 mg∙kg^−1^, 13,840 mg∙kg^−1^, and 10,436 mg∙kg^−1^ for the sewer sediments of catchment B, C, and D, respectively (Figure 9a). The sediments in catchment A also have abundant nitrogen, PO, and sulfides, and the average contents are 3462 mg∙kg^−1^, 672.74 mg∙kg^−1^, and 39.21 mg∙kg^−1^, respectively. For catchment B, the average content of TN, PO, and sulfides are 2214 mg∙kg^−1^, 453.61 mg∙kg^−1^, and 28.78 mg∙kg^−1^. For catchment C, the average content of TN, PO, and sulfides are 2617 mg∙kg^−1^, 657.41 mg∙kg^−1^, and 31.03 mg∙kg^−1^. For catchment D, the average content of TN, PO, and sulfides are 2133 mg∙kg^−1^, 672.74 mg∙kg^−1^, and 31.21 mg∙kg^−1^ (Figure 9b–d). Moreover, the contents of Pb and AOX in the sediments of catchment A and B are higher than those in the sediments of catchment C and D (Catchment A: Pb = 105.65 mg∙kg^−1^, AOX = 637.90 mg∙kg^−1^; Catchment B: Pb = 129.16 mg∙kg^−1^, AOX = 543.38 mg∙kg^−1^; Catchment C: Pb = 87.62 mg∙kg^−1^, AOX = 488.22 mg∙kg^−1^; Catchment D: Pb = 42.00 mg∙kg^−1^, AOX = 368.00 mg∙kg^−1^) (Figure 9e,f). According to Figure 9g, the cyanides content in the sediments is distributed from 0.040 mg∙kg^−1^ to 0.110 mg∙kg^−1^. The cyanides content in the sediments of catchment C and D is relatively stable, and the average content is 0.077 mg∙kg^−1^ and 0.075 mg∙kg^−1^, respectively. The cyanides content of the sediments of catchment A and B are distributed in a relatively wider range, with an average content of 0.064 mg∙kg^−1^ and 0.070 mg∙kg^−1^, respectively. The Zn concentrations in the sediments of catchment A and C are respectively 839.46 mg∙kg^−1^ and 1042.15 mg∙kg^−1^, which are much higher than the Zn concentrations of 387.29 mg∙kg^−1^ and 319.72 mg∙kg^−1^ in the sediments of catchment B and D (Figure 9h).

Based on the above analysis, it is definite that the distribution of contaminants in sediments in different spaces is inconsistent. RA in Longgang District brings more Hg and phosphorus pollution to sewer sediments than IA. Phosphorus, OM, Cu, and Ni in the sewer sediments mainly come from domestic sewage and industrial effluents, while cadmium is mainly brought by rainwater. Among the catchments of Longgang District, the distribution of many components in the sediments is quite different. The sediments of catchment C show the characteristics of high Zn content. The sediments in catchment A contain more nutrients (TK and TN) and more hazardous pollutants (PO, sulfides, and AOX) than in other catchments. The Pb content of some samples collected from catchment B is significantly higher than that of other catchments.

### 4.3. Difference between Sewer Sediment and Sewage Sludge

Although both sewer sediments and sewage sludge are closely related to wastewater, there are many differences in properties and composition between sewer sediments and sewage sludge. The relatively heavy suspended solids contained in wastewater are more inclined to deposit and form sewer sediments during transportation, while the relatively light suspended solids enter the wastewater treatment plant along with the wastewater and become sewage sludge after being treated. According to the previous study [24], sewage sludge usually has bulk densities that range from 0.5 g∙cm^−3^ to 1.1 g∙cm^−3^, which is significantly lighter than sewer sediments, and the densities of sewer sediments in this study ranged from 2.41 to 2.76 g∙cm^−3^. The moisture content of sewage sludge is extremely high, generally above 90%, while the moisture content of sewer sediment is only about 40%. The pH value of sewage sludge ranges from 5 to 8, while that of sewer sediments is relatively stably distributed from 6.54 to 7.06.

From the composition perspective, sewage sludge contains large amounts of OM, and the OM content of 30~55% in sewage sludge are reported [25,26], while the OM content of sewer sediment in this study is generally less than 10%. On the contrary, the ash content of sewer sediments (approximately 90%) is much higher than that of sewage sludge (approximately 30~50%). Compared with sewage sludge, the hazardous contaminants in sewer sediment are at a relatively low level. In these two kinds of municipal sludge, hazardous contaminants, especially heavy metals, showed similar distribution characteristics. In sewer sediments, heavy metals can be arranged in the following descending order based on their concentrations Zn > Cu > Cr > Pb > Ni > As > Cd > Hg, which is similar to the arrangement of heavy metals in sewage sludge in many previous studies [27,28,29,30].

### 4.4. Feasibility of Sewer Sediments Disposal

The predominant technical routes of sludge disposal in China mainly include land application, incineration, sanitary landfills, and building materials production [31,32,33]. It is estimated that approximately 29.3% of the sludge is disposed of via land application, followed by incineration (~26.7%), sanitary landfills (~20.1%), and building materials production (~15.9%) [34]. Sewer sediment is a new type of municipal sludge, and it is important to find a suitable technical route for its management.

#### 4.4.1. Land Application

Land application is the main method for municipal sludge management in China because it could make good use of nutrients (nitrogen, phosphorus, and potassium) and OM in the sludge. However, there are also many toxic components in the sludge, and the problem of contaminants toxicity for humans, animals, and plants should be considered carefully [35]. In China, GB/T 24600-2009 [36] and GB/T 23486-2009 [37] are implemented to control the quality of sludge used in land applications (Table S3 in Supplementary Information). According to these standards, the allowable limits of heavy metals in sludge are Cd < 5 mg∙kg^−1^, Hg < 5 mg∙kg^−1^, Pb < 300 mg∙kg^−1^, Cr < 600 mg∙kg^−1^, As < 75 mg∙kg^−1^, Ni < 100 mg∙kg^−1^, Cu < 800 mg∙kg^−1^, Zn < 2000 mg∙kg^−1^ when applied to acidic soils (pH < 6.5), and the allowable limits of heavy metals in sludge are Cd < 20 mg∙kg^−1^, Hg < 15 mg∙kg^−1^, Pb < 1000 mg∙kg^−1^, Cr < 1000 mg∙kg^−1^, As < 75 mg∙kg^−1^, Ni < 200 mg∙kg^−1^, Cu < 1500 mg∙kg^−1^, Zn < 4000 mg∙kg^−1^ when applied to neutral and alkaline soils (pH ≥ 6.5). When the sludge is used as a soil amendment to improve soil quality, the total nutrients content should be more than 1%, and the OM content should exceed 10%. For application in gardens or parks, the total nutrients content should not be less than 3%, and the OM content must surpass 25%. As shown in Table S2 in Supplementary Information, the concentration of Zn, Cu, Cr, Pb, Ni, As, Cd, and Hg in sewer sediments are 649.79 mg∙kg^−1^, 198.42 mg∙kg^−1^, 135.45 mg∙kg^−1^, 91.22 mg∙kg^−1^, 61.14 mg∙kg^−1^, 16.09 mg∙kg^−1^, 1.18 mg∙kg^−1^, 1.10 mg∙kg^−1^, respectively. In terms of the heavy metals concentration, the sewer sediments are meeting the requirement of land application. However, the total nutrients content of sewer sediment is 2.2%, which meets the quality of sludge used as a soil amendment but does not meet the quality of sludge used in gardens or parks. Moreover, the OM content in the sediment is only 5.2%, and samples with an OM content of more than 10% account for only 12.3%, which is far from the requirement. Although the concentration of hazardous contaminants in sewer sediments is under the allowable limits, the defect of insufficient OM content makes them inappropriate for land applications.

#### 4.4.2. Incineration and Co-Incineration

Recently, the proportion of municipal sludge treated by incineration is continuously increasing, which is why the viruses and pathogens could be eliminated and the volume of sludge also can be significantly reduced [38]. The standard of GB/T 24602-2009 [39] is implemented to control the quality of sludge used for incineration. According to the standard, both incineration and co-incineration require that the OM content in the sludge is not less than 50%. For incineration, the moisture content of the sludge cannot be higher than 50%, and the LHV is not less than 5000 kJ∙kg^−1^. For co-incineration, the moisture content of the sludge is not higher than 80%, and the LHV is not less than 3500 kJ∙kg^−1^. Also, the sulfides content in the sludge should be less than 2000 mg/kg to ensure that the incineration will not produce too much harmful gas [40]. Although the low moisture and sulfides content of sewer sediment is favorable (Figure 2b and Table S2 in Supplementary Information), its OM content and LHV are too poor to meet the requirements of incineration and co-incineration (Figure 3 and Figure 4). In addition, the average ash content of sewer sediment is 92.20% (Figure 4), which is much higher than that of sewage sludge (approximately 8%). Incineration has a very limited effect on the volume reduction of sewer sediment, and a large amount of solid waste remains. The above analysis shows that incineration is not suitable for the disposal of sewer sediments.

#### 4.4.3. Sanitary Landfills

In China, a large amount of municipal sludge is still disposed of via sanitary landfills. The standard of GB/T 23485-2009 [41] is implemented to control the quality of sludge for sanitary landfills (Table S3 in Supplementary Information). According to the standard, the pH value of sludge should be range from 5 to 10, and the moisture content should be no more than 60%, and the allowable limits of contaminants are as follow, PO < 3000 mg∙kg^−1^, VP < 40 mg∙kg^−1^, Cyanides < 10 mg∙kg^−1^, Cd < 20 mg∙kg^−1^, Hg < 25 mg∙kg^−1^, Pb < 1000 mg∙kg^−1^, Cr < 1000 mg∙kg^−1^, As < 75 mg∙kg^−1^, Ni < 200 mg∙kg^−1^, Cu < 1500 mg∙kg^−1^, Zn < 4000 mg∙kg^−1^, respectively. For sewer sediments, the pH value are range from 6.54 to 7.06, the moisture content is 39.3%, and the concentration of contaminants as follow, PO = 557.38 mg∙kg^−1^, VP = 0.38 mg∙kg^−1^, Cyanides = 0.07 mg∙kg^−1^, Cd = 1.18 mg∙kg^−1^, Hg = 1.10 mg∙kg^−1^, Pb = 91.22 mg∙kg^−1^, Cr = 135.45 mg∙kg^−1^, As = 16.09 mg∙kg^−1^, Ni = 61.14 mg∙kg^−1^, Cu = 198.42 mg∙kg^−1^, and Zn = 649.79 mg∙kg^−1^, respectively. The concentration of contaminants in the sewer sediment all meets the allowable limits. The properties of sewer sediment also meet the requirements, which means the sewer sediment can be managed by landfill. However, according to previous studies on municipal sludge landfills [42,43,44], the surrounding environment of the landfill site still has the potential risk of being polluted by sewer sediment leachate. In addition, due to the limited land resources and the advocacy of a zero-waste strategy, sanitary landfills are gradually being restricted in Shenzhen.

#### 4.4.4. Building Materials

The management of municipal sludge via the production of building materials is attracting increasing attention in China. According to previous studies, municipal sludge can be used to prepare brick [45,46], ceramsite [47,48,49], and concrete [50]. For brick making, the quality of sludge should meet the requirements of GB/T 25031-2010 [51] (Table S3 in Supplementary Information). According to the standard, the allowable limits of contaminants are as follow, PO < 3000 mg∙kg^−1^, VP < 40 mg∙kg^−1^, Cyanides < 10 mg∙kg^−1^, Cd < 20 mg∙kg^−1^, Hg < 5 mg∙kg^−1^, Pb < 300 mg∙kg^−1^, Cr < 1000 mg∙kg^−1^, As < 75 mg∙kg^−1^, Ni < 200 mg∙kg^−1^, Cu < 1500 mg∙kg^−1^, Zn < 4000 mg∙kg^−1^, respectively. Although brick making has stricter requirements on the quality of sludge than landfill in terms of Hg and Pb concentration, the content of toxic substances in the sewer sediment is still within the allowable limits. In addition, the high ash content of sewer sediments is favorable for brick making, which is required that not less than 50% in the standard. Except for brick making, ceramsite preparation may also be a good choice for the utilization of sewer sediments, which can be satisfied by various raw materials. Moreover, the heavy metals contained in sewer sediments can be immobilized in the ceramsite [48,49]. These approaches can not only avoid the environmental risks caused by sewer sediments accumulation but also produce valuable building material products.

## 5. Conclusions

This study characterizes the sewer sediments of CSS in Longgang District, Shenzhen. In addition, a bivariate correlation analysis of variables related to sewer sediments was carried out, and the feasibility of disposal was discussed. The main conclusions drawn are as follow:

(1) Sewer sediment is a type of municipal sludge with high bulk density and relatively low moisture content. Sand is the main particle presence in sewer sediments and followed by silt and clay. Compared with sewage sludge, sewer sediments contain less OM and nutrients, have relatively low heat production potential, and still leave a large amount of fly ash after incineration. The composition of the sediment is complicated and contains many pollutants, including heavy metals, sulfides, PO, AOX, cyanides, and VP. Heavy metals can be arranged in the following descending order based on their concentrations Zn > Cu > Cr > Pb > Ni > As > Cd > Hg.

(2) The distribution of contaminants in sewer sediments in different spaces is inconsistent. Compared to IA, the sewer sediments in RA contain higher concentrations of Hg and phosphorus. The sediment in the sewage conduit contains more OM, phosphorus, Cu, and Ni, and the sediment in the rainwater conduit contains a higher concentration of Cd. In addition, the sediments produced in different catchments also show huge differences in the content of contaminants. We may be able to classify sewer sediments based on these spatial distribution characteristics, to more specifically dispose of these wastes and reduce their environmental risks.

(3) The low OM content limits the use of sewer sediments in land applications. Incineration is also not suitable for the disposal of sewer sediments, due to the poor LHV and high ash content. Although it can meet the requirements of local landfill standards, the complex pollutant composition in sewer sediments still poses the risk of polluting the surrounding environment. The management of sewer sediments via the production of building materials is a promising technical route that can avoid the migration of heavy metals and produce valuable products.

## Data Availability

Not applicable.

## Supplementary Materials

The following are available online at https://www.mdpi.com/article/10.3390/ijerph18147687/s1, Table S1: Spatial properties of sewer sediment samples, Table S2: Hazardous contaminants concentration of sewer sediment samples, Table S3: Standards of municipal sludge disposal.
