# Supplementary material for: Characterization, Spatial Variation and Management Strategy of Sewer Sediments Collected from Combined Sewer System: A Case Study in Longgang District, Shenzhen
Source: Int J Environ Res Public Health. 2021 Jul 20;18(14):7687. doi: 10.3390/ijerph18147687 (PMC8303206; doi:10.3390/ijerph18147687)
Supplement: Supplementary file 1 [file ijerph-18-07687-s001.zip › ijerph-1284556-supplementary.pdf]

Title

Sewer sediments of combined sewer system in Longgang district, Shenzhen: characterization, spatial variation and management

Authors

Yongpeng Luo [a], Shenxu Bao [a,*], Siyuan Yang [a], Yimin Zhang [a,b], Yang Ping [c], Chao Lin [c], Pan Yang [d]

Affiliations

[a] School of Resources and Environmental Engineering, Wuhan University of Technology, Wuhan 430070, PR China.

[b] State Environmental Protection Key Laboratory of Mineral Metallurgical Resources Utilization and Pollution Control, Wuhan University of Science and Technology, Wuhan 430081, PR China.

[c] Shenzhen Water Planning and Design Institute Co., Ltd, Shenzhen 518116, PR China.

[d] Department of Civil and Environmental Engineering, University of Illinois at Urbana Champaign, 205 N Mathews Ave, Urbana, IL 61820, USA

***Corresponding author**

Email: sxbao@whut.edu.cn

Table S1 Spatial properties of sewer sediment samples

Sample number	Land use	Conduit type	Catchment
1	RA	BC	D
2	RA	BC	D
3	RA	BC	D
4	RA	BC	A
5	RA	BC	A
6	RA	BC	B
7	RA	BC	B
8	RA	BC	B
9	RA	BC	C
10	RA	BC	C
11	RA	SC	D
12	RA	SC	D
13	RA	SC	D
14	RA	SC	A
15	RA	SC	A
16	RA	SC	A
17	RA	SC	B
18	RA	SC	B
19	RA	SC	B
20	RA	SC	C
21	RA	SC	C
22	RA	SC	C
23	RA	SC	C

24	RA	SSC	D
25	RA	SSC	D
26	RA	SSC	D
27	RA	SSC	A
28	RA	SSC	A
29	RA	SSC	A
30	RA	SSC	B
31	RA	SSC	B
32	RA	SSC	B
33	RA	SSC	C
34	RA	SSC	C
35	RA	SSC	C
36	RA	CC	C
37	RA	CC	C
38	RA	CC	C
39	RA	CC	C
40	IA	BC	D
41	IA	BC	D
42	IA	BC	A
43	IA	BC	A
44	IA	BC	B
45	IA	BC	B
46	IA	BC	C
47	IA	SC	D
48	IA	SC	D
49	IA	SC	A

50	IA	SC	A
51	IA	SC	B
52	IA	SC	B
53	IA	SC	B
54	IA	SC	C
55	IA	SSC	D
56	IA	SSC	D
57	IA	SSC	A
58	IA	SSC	A
59	IA	SSC	B
60	IA	SSC	B
61	IA	SSC	B
62	IA	SSC	B
63	IA	SSC	C
64	IA	SSC	C
65	IA	SSC	C
66	IA	CC	D
67	IA	CC	D
68	IA	CC	D
69	IA	CC	A
70	IA	CC	A
71	IA	CC	A
72	IA	CC	A
73	IA	CC	A

RA = Residential area

IA = Industrial area

BC = Box culverts
SSC = Storm sewer conduits
SC = Sewage conduits
CC = Combined conduits

Table S2 Hazardous contaminants concentration of sewer sediment samples

Sample number	Cd (mg/kg)	Hg (mg/kg)	Pb (mg/kg)	Cr (mg/kg)	As (mg/kg)	Ni (mg/kg)	Zn (mg/kg)	Cu (mg/kg)	Sulfides (mg/kg)	AOX (mg/kg)	PO (mg/g)	VP (mg/kg)	Cyanides (mg/kg)
1	0.38	0.21	49.51	96.49	32.30	29.86	228.97	85.52	32.0	117.52	0.43	0.3	0.06
2	0.51	0.10	48.63	60.91	7.19	25.62	435.01	49.41	26.2	75.61	0.66	0.7	0.08
3	0.89	0.50	51.90	118.00	13.80	58.00	453.00	336.00	32.1	368.10	0.40	0.3	0.08
4	0.83	0.54	140.00	164.60	27.70	130.59	810.29	390.67	41.9	432.57	0.71	0.4	0.05
5	0.67	0.28	139.00	209.34	23.20	146.87	684.34	373.10	54.7	427.80	0.55	0.4	0.05
6	0.27	0.04	47.00	42.00	15.60	20.00	211.00	30.00	26.6	56.60	0.44	0.3	0.08
7	0.73	0.58	57.80	109.00	8.43	45.00	463.00	60.00	29.3	89.30	0.55	0.6	0.06
8	0.58	0.25	40.30	52.00	6.97	31.00	552.20	50.00	36.6	86.60	0.45	0.3	0.08
9	0.44	2.94	71.90	74.00	30.80	34.00	503.00	1190.00	32.9	1222.90	0.61	0.6	0.07
10	0.37	0.76	54.09	142.93	11.30	53.74	437.57	84.87	27.0	111.87	0.45	0.5	0.07
11	0.30	4.14	39.60	162.38	24.00	54.37	309.20	118.11	27.4	145.51	0.62	<0.1	0.06
12	1.21	1.25	51.74	85.19	13.90	65.94	565.76	125.63	27.3	152.93	0.72	<0.1	0.07
13	0.60	0.68	64.62	78.58	17.50	91.14	531.75	706.70	37.2	743.90	0.57	0.2	0.08
14	0.31	0.70	74.76	97.87	18.70	47.90	280.46	132.52	37.3	169.82	0.71	0.5	0.06
15	1.12	0.22	143.00	70.00	17.60	65.00	298.00	77.00	35.8	112.80	0.80	0.2	0.08
16	1.11	0.63	82.16	298.64	17.40	62.36	1710.00	237.95	37.3	275.25	0.82	0.1	0.06
17	1.80	1.12	77.90	78.00	6.87	43.00	517.00	105.00	34.5	139.50	0.84	0.4	0.07
18	1.52	11.30	113.00	74.00	10.70	39.00	971.00	64.00	29.8	93.80	0.56	<0.1	0.07
19	4.04	0.07	254.00	35.00	4.79	12.00	164.00	24.00	30.2	54.20	0.25	0.8	0.08
20	0.59	0.18	71.00	72.00	25.10	32.00	623.00	119.00	25.1	144.10	0.64	0.3	0.07
21	0.93	4.95	337.00	94.00	58.20	46.00	712.00	155.00	24.4	179.40	0.74	0.2	0.07
22	0.47	0.07	32.20	39.00	12.80	35.00	383.95	62.00	24.0	86.00	0.73	0.5	0.07

23	0.41	0.28	45.40	170.36	8.91	83.88	369.21	116.77	45.6	162.37	0.77	0.4	0.09
24	0.45	0.38	39.32	115.66	21.00	40.03	275.30	73.00	28.9	101.90	0.43	<0.1	0.07
25	1.19	1.31	54.84	87.53	15.10	70.32	581.09	122.52	32.8	155.32	0.31	0.3	0.06
26	0.53	0.10	30.46	58.92	19.40	25.41	102.73	33.91	28.8	62.71	0.22	0.4	0.06
27	0.38	1.19	98.40	110.73	10.30	49.80	391.74	132.64	35.0	167.64	0.48	0.2	0.07
28	0.86	0.65	206.00	95.67	19.40	57.71	383.42	90.39	43.5	133.89	0.50	0.5	0.08
29	0.46	0.12	159.00	65.56	16.40	43.70	177.20	53.53	49.2	102.73	0.64	0.1	0.06
30	0.41	0.10	29.80	40.00	4.68	14.00	339.00	33.00	28.1	61.10	0.36	0.7	0.08
31	18.28	0.13	1130.00	87.00	7.38	25.00	444.00	68.00	28.6	96.60	0.31	0.3	0.07
32	0.10	0.06	42.10	21.00	3.72	12.00	115.00	10.00	24.8	34.80	0.31	0.5	0.06
33	1.08	1.16	110.00	177.00	10.00	51.00	1000.00	640.00	31.6	671.60	0.48	<0.1	0.11
34	0.97	4.77	40.50	44.00	14.90	39.00	565.00	110.00	30.9	140.90	1.07	<0.1	0.06
35	0.55	1.29	80.48	82.22	31.60	35.57	318.33	90.74	34.7	125.44	0.73	0.6	0.08
36	0.43	1.30	60.68	98.60	16.90	49.34	559.77	328.13	33.8	361.93	0.36	0.2	0.07
37	0.20	0.13	27.31	75.51	0.56	28.17	172.10	47.93	29.6	77.53	0.23	0.2	0.07
38	1.17	1.53	63.63	320.64	8.66	103.95	818.47	220.14	29.3	249.44	0.82	0.5	0.07
39	1.08	4.62	29.76	16.95	11.60	38.34	613.25	136.94	37.5	174.44	1.10	0.5	0.08
40	0.33	0.10	36.70	48.49	15.80	32.68	143.43	55.08	42.9	97.98	0.34	0.4	0.09
41	0.34	0.09	37.86	53.35	17.30	36.26	146.20	52.28	29.0	81.28	0.21	<0.1	0.08
42	0.85	0.65	84.40	294.75	15.70	128.36	664.64	249.66	45.6	295.26	0.89	0.4	0.09
43	0.48	0.35	71.04	184.06	18.70	116.44	419.02	238.22	42.2	280.42	0.53	0.2	0.07
44	0.31	0.04	49.60	45.00	7.74	22.00	309.00	71.00	23.4	94.40	0.32	0.2	0.06
45	0.37	0.07	61.60	24.00	12.70	22.00	204.00	19.00	27.6	46.60	0.43	0.3	0.05
46	1.08	2.02	129.00	336.00	23.20	78.00	2080.00	284.00	29.8	313.80	0.81	0.6	0.07
47	1.02	1.08	84.39	799.74	21.10	326.40	580.42	153.84	34.4	188.24	0.65	0.1	0.08
48	1.63	0.09	18.46	61.57	16.00	105.02	223.06	119.57	32.0	151.57	0.39	<0.1	0.09

49	4.05	1.48	91.47	240.45	12.60	120.05	682.69	339.53	32.6	372.13	0.59	0.4	0.07
50	0.62	0.44	42.55	86.83	17.40	58.20	1140.00	91.08	34.3	125.38	0.83	0.1	0.06
51	1.02	0.49	157.00	656.00	7.61	81.00	844.00	474.00	34.3	508.30	0.77	0.4	0.07
52	0.19	0.42	41.80	26.00	3.56	12.00	197.00	65.00	28.5	93.50	0.34	0.6	0.08
53	1.36	3.79	57.50	121.00	10.20	39.00	805.00	117.00	24.3	141.30	0.83	0.3	0.08
54	0.67	0.05	56.00	53.00	26.30	22.00	294.00	46.00	32.6	78.60	0.26	0.2	0.09
55	0.34	0.07	29.55	56.38	19.80	18.87	121.16	25.81	32.2	58.01	0.34	0.4	0.08
56	0.18	0.04	28.99	37.02	14.40	20.03	40.21	15.40	30.8	46.20	0.32	0.4	0.07
57	0.62	0.31	136.00	66.39	21.10	33.74	452.16	1067.40	40.3	1107.70	0.52	<0.1	0.07
58	0.69	0.21	92.93	352.64	18.80	169.55	693.70	345.03	45.0	390.03	0.59	0.3	0.08
59	0.04	0.08	35.60	11.00	1.92	10.00	108.00	21.00	28.3	49.30	0.19	0.4	0.06
60	0.68	0.10	55.30	53.00	7.16	24.00	505.00	52.00	26.7	78.70	0.69	0.7	0.07
61	0.03	0.07	28.40	15.00	2.93	13.00	102.00	21.00	25.3	46.30	0.22	0.5	0.06
62	0.10	0.07	46.10	22.00	4.58	13.00	121.00	29.00	31.1	60.10	0.31	0.5	0.08
63	3.84	9.06	42.80	111.00	44.10	48.00	739.00	160.00	29.4	189.40	0.76	0.8	0.08
64	0.57	4.78	63.40	97.00	19.60	42.00	7130.00	102.00	29.2	131.20	0.58	<0.1	0.08
65	8.71	0.47	262.00	171.00	25.70	52.00	1440.00	233.00	31.2	264.20	0.69	<0.1	0.09
66	0.73	0.06	28.74	112.16	19.70	220.33	379.63	205.19	30.6	235.79	0.45	0.1	0.08
67	1.55	0.21	24.93	95.35	19.90	204.22	353.59	163.64	27.7	191.34	0.43	0.1	0.07
68	1.26	0.08	29.20	81.94	27.90	137.78	284.40	139.04	30.2	169.24	0.43	0.3	0.09
69	1.24	0.53	84.13	595.20	19.00	83.40	3120.00	872.98	37.0	909.98	0.92	0.3	0.06
70	1.03	0.65	110.00	251.06	13.60	87.97	753.57	354.23	32.6	386.83	0.75	0.5	0.08
71	0.34	0.11	80.59	229.49	19.40	18.74	707.06	877.53	33.7	911.23	0.54	<0.1	0.04
72	1.15	0.33	92.50	398.28	11.10	46.81	2060.00	301.91	34.8	336.71	0.71	0.4	0.04
73	0.60	1.34	79.44	180.76	12.80	84.53	521.48	239.48	33.6	273.08	0.70	0.2	0.05

Table S3 Standards of municipal sludge disposal

Parameters	Allowable limits					
	GB/T 24600-2009 (Soil amendment)		GB/T 23486-2009 (Gardens or parks)		GB/T 23485-2009 (Sanitary landfill)	GB/T 25031-2010 (Making brick)
	Acidic soil (pH<6.5)	Neutral and alkaline soils (pH⩾6.5)	Acidic soil (pH<6.5)	Neutral and alkaline soils (pH⩾6.5)		
pH	5.5~10		6.5~8.5	5.5~7.8	5~10	5~10
Moisture (%)	<65		<40		<60	<40
Total nutrients (TK+TN+TP, %)	⩾1		⩾3		-	-
Organic matter (%)	⩾10		⩾25		-	-
Petroleum oil (mg/kg)	3000		3000		3000	3000
AOX (mg/kg)	500		500		-	-
Volatile phenols (mg/kg)	40		-		40	40
Cyanides (mg/kg)	10		-		10	10
Cd (mg/kg)	5	20	5	20	20	20
Hg (mg/kg)	5	15	5	15	25	5
Pb (mg/kg)	300	1000	300	1000	1000	300
Cr (mg/kg)	600	1000	600	1000	1000	1000
As (mg/kg)	75	75	75	75	75	75
Ni (mg/kg)	100	200	100	200	200	200
Cu (mg/kg)	800	1500	800	1500	1500	1500
Zn (mg/kg)	2000	4000	2000	4000	4000	4000
